# Supplementary material for: The effects of luck perception on consumer variety-seeking
Source: PLoS One. 2025 Jun 11;20(6):e0324896. doi: 10.1371/journal.pone.0324896 (PMC12157096; doi:10.1371/journal.pone.0324896)
Supplement: Attachment — Submitted filename: Modification instructions.docx [file pone.0324896.s003.docx]

# Modification instructions

**Dear Reviewers:**

I would like to thank you sincerely for your valuable comments on this paper, which were very professional and pertinent, pointing out shortcomings and giving suggestions for revision, which have been very enlightening. Your suggestions have been very helpful and we. The authors have followed your comments to read and revise the whole text again and again and to explain it in detail.

**Reviewer #1**

General Assessment
1. Originality and Contribution to the Field. The manuscript explores the under-researched area of luck perception in consumer behavior, offering valuable insights into its psychological underpinnings and practical implications. Recommendation: Highlight the novelty of examining luck perception in the context of variety-seeking and its practical relevance to marketing strategies. Discuss how these findings fill gaps in existing research on consumer behavior and psychological motivations.
**Revision note**: Thank you very much for your suggestions.

We have summarized the research significance of this paper as follows:

“In this paper, the influence of luck on variety-seeking was explored in depth through experimental research methods. The main research questions include the direct influence of luck on variety-seeking, the mechanism of novelty-seeking motivation in the effect of luck on variety-seeking, and the boundary conditions need for cognitive closure. The findings offer innovative insights into the influence of luck in the field of consumption, broadening the scope of research on luck effects in product or brand decision-making. Furthermore, they delve into the psychological effects of luck, providing valuable theoretical support for people to gain a scientific understanding of luck. From a practical viewpoint, this paper’s findings will shed light on the expansion of product lines and the launch of new brands and provide guidance for enterprises to implement precision marketing as well as luck marketing.”

2. Clarity and Structure. The manuscript is generally well-organized, but the dense presentation of experimental methods and statistical analyses may be challenging for readers unfamiliar with these techniques. Recommendation: Simplify the descriptions of experimental designs and statistical methods. Consider adding summaries or diagrams to make complex concepts more accessible.
**Revision note**: Thank you very much for your suggestions. This paper is written in accordance with the norms for psychological experiments, employing standardized terminology in the experimental section. If the processes and designs are overly simplified, readers may struggle to comprehend the entire experimental procedure, rendering it unhelpful as a reference for similar experimental designs in the future.

3. Theoretical Framework. The manuscript presents a solid theoretical foundation, but the discussion of novelty-seeking motivation and the need for cognitive closure could be more comprehensive. Recommendation: Provide a deeper discussion of novelty-seeking and cognitive closure theories. Explain their relevance to consumer behavior and their integration with luck perception.
**Revision note**: Thank you very much for your questions and suggestions.

We have reorganized the logical reasoning of the hypothesis to make the explanation of novelty-seeking motivation and the need for cognitive closure more comprehensive. The specifics are as follows:

“Research on luck suggests that consumers who feel lucky become more energetic and possess more preferences for unfamiliar or novel choices. For example, consumers who feel lucky are more likely to use online banking, rather than a physical bank, to deposit money (Jiang et al., 2009). In financial markets, consumers who feel lucky have a higher interest in sudden and unfamiliar market information; they are more willing to pay for random shock signals in the lottery market (Gao et al., 2021). It is evident that luck motivates consumers and develops their interest in and desire for novel and unknown things. Novelty seeking is the innate tendency of consumers to show pleasure or excitement for novel stimuli (Thomas-Walters, 2021).

Based on the above analysis, we argue that consumers who feel lucky possess a strong motivation to pursue novelty.

The motivation for novelty seeking is one of the important factors influencing individual behavior. It represents consumers' innate tendency to exhibit pleasure or excitement towards novel stimuli, and serves as one of the driving forces for consumers to actively pursue new experiences (Nguyen et al., 2020). This is manifested in two aspects: intrinsic novelty seeking is the individual's desire for novel and exciting stimuli; extrinsic novelty seeking is manifested in the individual's actual behavior to obtain new stimuli. Therefore, we believe that the mechanism of luck’s influence on variety-seeking emerges from consumer novelty-seeking motivation. This is because variety-seeking is an expression of consumers’ search for novelty and pursuit of maximizing psychological utility, which can satisfy consumers’ novelty needs (Cao & Wang 2018). If consumers repeatedly choose a product, their stimulation for the product becomes weaker and fails to satisfy their novelty needs. on the other hand, found that when consumers have strong novelty-seeking motivation, they will change their routine purchasing behaviors, such as choosing new and unfamiliar products or brands. Meanwhile, when their novelty-seeking motivation is weaker, they will continue to maintain routine buying behavior (Sahni & Gupta, 2019).”

“Cognitive closure needs, as a motivational factor influencing individuals' ambiguous decision-making in uncertain situations, are composed of dimensions such as cognitive structuring, ambiguity aversion, decision decisiveness, expectation predictability, and psychological closure. Cognitive closure needs are both personality traits and context-dependent, and can be aroused by time pressure, psychological fatigue, environmental noise, and task attractiveness. Individuals with higher cognitive closure needs often follow the principles of decision immediacy and decision continuity, and have a strong tendency towards grasping and freezing. Cognitive closure needs affect individuals' attitudes towards ambiguity and information processing methods. Those with higher cognitive closure needs have lower ambiguity tolerance and are more prone to heuristic information processing; whereas consumers with lower cognitive closure needs have higher ambiguity tolerance and are more likely to engage in analytical information processing. Individuals with high cognitive closure needs rely more on conventions or stereotypes during information processing, less on information search, and are prone to forming larger decision biases; whereas those with low cognitive closure needs have higher ambiguity tolerance during information processing, rely more on information search, and therefore make more accurate decisions. These research conclusions have been widely tested in the fields of social judgment, team interaction, consumer behavior, negotiation, and politics.”

4. Methodology. The experimental designs are robust, but some aspects of the methodology, such as participant selection, priming techniques, and statistical assumptions, require further elaboration. Recommendation: Clearly explain the criteria for participant recruitment and how cultural factors might influence responses to luck perception. Justify the choice of priming methods and discuss potential biases.
**Revision note**: Thank you very much for your questions and suggestions. We have made further improvements to address the deficiencies in this area. The specifics are as follows:

Experiment 1：“The subjects are required to have independent consumption experience. The experiment used a single factor analysis method (type of priming: lucky vs. unlucky vs. control), and participants were randomly assigned to the lucky (63), unlucky (75), and control (127) groups.”

Experiment 2：“We recruit participants using the credamo research platform (www.credamo.com), and require them to have independent consumption experience. one hundred and forty-four consumers participated in the experiment, with a mean age of 35.32 years (SD = 4.93); 56 were male, accounting for 38.89%. A single factor (type of priming: lucky vs. unlucky) experiment was used, and participants were randomly assigned to any group. There were 72 each in the lucky and unlucky groups.”

Experiment 3：“The subjects are required to have independent consumption experience. a total of 224 customers participated in the experiment, with a mean age of 36.99 years (SD = 6.92); 67 or 29.91% were male. They were randomly assigned to the lucky group (114) and the unlucky group (110) and asked to participate in two ostensibly unrelated experiments. The first was a questionnaire on traits needed for cognitive closure, and the latter was a consumer choice experiment.”

In addition, the methods for initiating luck perception all refer to mature methods（Jiang et al., 2009; Li, 2021; Zhao & Zhong, 2022), which have been applied in the literature. Therefore, I believe there is no need to further discuss the feasibility of these methods.

**Reference**

Jiang, Y., Cho, A., & Adaval, R. (2009). The unique consequences of feeling lucky: Implications for consumer behavior. *Journal of Consumer Psychology*, 19(2), 171-184.

Li, H. (2021). Confidence charms: How superstition influences overconfidence bias in Han and the Qiang ethnic minority Chinese. *The Journal of Psychology*, 155(5), 473-488.

Zhao, J., & Zhong, H. (2022). The licensing effect of luck: The influence of perceived luck on green consumption intention. *Current Psychology*, 42(15), 1-15.

5. Operationalization of Key Variables. Key variables like luck perception, novelty-seeking motivation, and need for cognitive closure are defined but require more detail on their measurement and validation. Recommendation: Provide detailed descriptions of scales used to measure these variables, including their reliability and validity. Explain how the experimental manipulations were verified.

**Revision note**: Thank you very much for your questions and suggestions. According to existing research, researchers generally only report reliability, while reporting on validity is relatively rare. In this experiment, there is only one scale, so it is impossible to conduct a validity analysis.

In addition, this experiment utilized mature priming methods, and the results have proven these methods to be feasible. We tested luck perception in three experiments and found that the experimental group had a higher perception of luck compared to the control group, indicating that the experimental priming was effective.

6. Results Interpretation. The results are well-presented, but their practical implications are underexplored. Recommendation: Discuss the real-world applications of these findings, such as how marketers can use luck perception to design campaigns that encourage variety-seeking. Provide specific examples of potential strategies.
 “Luck is increasingly being used by businesses as an actionable marketing element to influence consumer behavior. These firms want to influence consumers’ consumption decisions by improving their luck perception. Therefore, This paper’s findings have managerial implications for business managers or marketers.

Firstly, the findings have implications for firms expanding their product lines and launching new brands. When enterprises launch new products or brands, enterprise managers or marketers can consider altering consumers' luck perception to influence their consumption decisions. For instance, they can incorporate lucky numbers or symbols on product packaging or during advertising campaigns. When promoting products or brands offline, they can utilize lucky projection techniques or lucky draws to stimulate consumers' luck perception, fostering a greater variety of demands and enhancing their preference for new products or brands, thereby achieving market promotion for new products and brands.

Secondly, the research conclusions assist enterprises in implementing precise marketing. Enterprise managers or marketers can utilize big data technology, such as by simplifying the cognitive closure needs scale, to simply inquire about consumers' cognitive closure needs and identify their personality traits, thereby tracking and identifying consumers with high cognitive closure needs. When facing these consumers, enterprise managers or marketers can push luck-related information to them while they browse products, stimulating their sense of luck, increasing their variety-seeking behavior, and encouraging them to purchase more different products, thereby increasing the sales volume of the enterprise.”

7. Moderation and Mediation Effects. While the moderating and mediating effects are statistically significant, their broader implications are not fully discussed. Recommendation: Elaborate on how the need for cognitive closure influences the relationship between luck perception and variety-seeking. Discuss the implications of novelty-seeking motivation as a mediator in consumer decision-making.
We discussed in detail how the need for cognitive closure affects the relationship between luck perception and variety-seeking, as well as the mediating role of novelty-seeking motivation in consumer decision-making.

***The mediating role of novelty-seeking motivation***

Research on luck suggests that consumers who feel lucky become more energetic and possess more preferences for unfamiliar or novel choices. For example, consumers who feel lucky are more likely to use online banking, rather than a physical bank, to deposit money (Jiang et al., 2009). In financial markets, consumers who feel lucky have a higher interest in sudden and unfamiliar market information; they are more willing to pay for random shock signals in the lottery market (Gao et al., 2021). It is evident that luck motivates consumers and develops their interest in and desire for novel and unknown things. Novelty seeking is the innate tendency of consumers to show pleasure or excitement for novel stimuli (Thomas-Walters, 2021).

Based on the above analysis, we argue that consumers who feel lucky possess a strong motivation to pursue novelty.

The motivation for novelty seeking is one of the important factors influencing individual behavior. It represents consumers' innate tendency to exhibit pleasure or excitement towards novel stimuli, and serves as one of the driving forces for consumers to actively pursue new experiences (Nguyen et al., 2020). This is manifested in two aspects: intrinsic novelty seeking is the individual's desire for novel and exciting stimuli; extrinsic novelty seeking is manifested in the individual's actual behavior to obtain new stimuli. Therefore, we believe that the mechanism of luck’s influence on variety-seeking emerges from consumer novelty-seeking motivation. This is because variety-seeking is an expression of consumers’ search for novelty and pursuit of maximizing psychological utility, which can satisfy consumers’ novelty needs (Cao & Wang 2018). If consumers repeatedly choose a product, their stimulation for the product becomes weaker and fails to satisfy their novelty needs. on the other hand, found that when consumers have strong novelty-seeking motivation, they will change their routine purchasing behaviors, such as choosing new and unfamiliar products or brands. Meanwhile, when their novelty-seeking motivation is weaker, they will continue to maintain routine buying behavior (Sahni & Gupta, 2019).

In sum, luck perception can give consumers a power resource that enhances their novelty-seeking motivation, and variety-seeking can satisfy their novelty-seeking needs. Therefore, luck affects consumer variety-seeking, and novelty-seeking motivation is the mediating mechanism.

**Hypothesis 2**: Novelty-seeking motivation mediates the influence of luck perception on consumer variety-seeking.

***Moderating Role of Need for Cognitive Closure***

Research has shown that relative to consumers with low need for cognitive closure, consumers with high need for cognitive closure are cognitively lazy, tend to adopt heuristic, simplified and top-down information processing, are prone to make decisions based on some marginal or peripheral cues, and their behaviors are susceptible to proximate cause effects (Wronska et al., 2019). Liu et al. (2018) argued that in an uncertain environment, in order to form conclusions quickly, consumers with high need for cognitive closure rely on simple, unattached rules, and take recent events as the basis for decision-making. Thus, recent event cues have a significant effect on the behavioral decisions of consumers with high need for cognitive closure. For consumers with a high need for cognitive closure, if they encounter cues of luck, they are easily stimulated by these cues, which means they are prone to experiencing a sense of luck. Consequently, they tend to use this perceived luck as a guide for variety-seeking choices and are inclined to engage in variety-seeking behaviors. Therefore, for consumers with a high need for cognitive closure, luck perception has a significant impact on their preference for variety-seeking.

On the other hand, consumers with low need for cognitive closure are motivated to avoid cognitive closure and are easily affected by potential cues (such as emotions and stable psychological cognition). They seek alternative cues as the basis for decision-making. Therefore, consumers with low need for cognitive closure are cognitively hardworking, and recency effect will not have a strong influence on their behavioral decision making (Sharifi, 2019). When faced with variety-seeking choices consumption scenarios, consumers with low need for cognitive closure rely on central, stable mental cues to make decisions, and their variety-seeking preferences do not change significantly, whether they are stimulated by luck or not. Therefore, for consumers with low need for cognitive closure, the influence of luck perception on the preference for variety-seeking does not undergo significant changes。

In summary, we believe that consumers with different levels of need for cognitive closure possess different recency cause effects. For consumers with high need for cognitive closure, when making variety-seeking choices, they are susceptible to pre-decision luck stimuli or experiences due to the strong influence of recency effects on behavioral decisions. For consumers with low need for cognitive closure, when making variety-seeking choices, the pre-decision luck stimulus or experience will not have a strong influence on their variety-seeking due to the weak influence of recency effects on behavioral decision making.

**Hypothesis 3**: The effect of luck perception on variety-seeking is only signifcant among the consumers with high need for cognitive closure, but insignifcant for consumers with low need for cognitive closure.

**Reviewer #2**

1. Title and Abstract：

Title Comment: The title is clear and informative but could emphasize the experimental nature of the study. Recommendation: Consider revising the title to reflect the experimental approach (e.g., "Experimental Evidence on the Role of Luck Perception in Consumer Variety-Seeking").
**Revision note**: We searched for research papers related to luck perception, but none of them mentioned experimental methods in their titles, so we still believe the current title is more accurate.

**Reference**

Luo, Z., & Wu, Y. (2022). The influence of perception of luck on consumers’ behavior and its theoretical explanation. *Advances in Psychological Science*, 30(2), 464-476.

Pontes, N., & Williams, L. K. (2021). Feeling red lucky? The interplay between color and luck in gambling settings. *Psychology & Marketing*, 38(1), 43-55.

Young, M. J., Chen, N., & Morris, M. W. (2009). Belief in stable and fleeting luck and achievement motivation. *Personality and Individual Differences*, 47(2), 150-154.

Zhao, J., & Zhong, H. (2022). The licensing effect of luck: The influence of perceived luck on green consumption intention. *Current Psychology*, 42(15), 1-15.

Zhou, K., Tang, H., Sun, Y., Huang, G. H., Rao, L. L., Liang, Z. Y., & Li, S. (2012). Belief in luck or in skill: Which locks people into gambling?. *Journal of Gambling Studies*, 28(3), 379-391.

Comment: The abstract effectively summarizes the study but lacks emphasis on practical implications. Recommendation: Include a brief discussion of how these findings could inform marketing practices or consumer engagement strategies.
**Revision note**: We have summarized the practical significance and guidance for enterprises of this paper in the abstract.

“This study provides theoretical support for understanding luck scientifically and enlightens and guides companies to expand product lines, present new brands, and implement precision marketing.”

2. Introduction
Comment : The introduction provides a strong rationale for the study but could better contextualize the research within broader consumer behaviour literature.
Recommendation: Add a discussion of how this research complements or extends existing studies on psychological influences in consumer decision-making.
**Revision note**: We explored the theoretical significance of the research findings presented in this paper.

“The findings offer innovative insights into the influence of luck in the field of consumption, broadening the scope of research on luck effects in product or brand decision-making. Furthermore, they delve into the psychological effects of luck, providing valuable theoretical support for people to gain a scientific understanding of luck.”

3. Literature Review
Comment: The review covers key concepts but does not fully integrate them into a cohesive framework. Recommendation: Expand on the relationships between luck perception, novelty-seeking motivation, and variety-seeking. Include additional references to recent studies in consumer psychology.
**Revision note**: We have conducted a more comprehensive exploration of the relationship among luck perception, novelty-seeking motivation, and variety-seeking.

***Luck Perception, Novelty-seeking Motivation and Variety-seeking***

People use the essence of luck, as an external tool, to eliminate uncertainty in the environment. This external tool is luck that makes individuals feel that they are with powerful people (Damisch et al., 2010). According to the psychological power theory of luck, luck can be perceived as an extended power of the individual that can influence events in their favor (Young et al., 2009). Thus, luck becomes a power resource for consumers, allowing them to develop a positive cognitive bias that can make them overestimate their self-control in event processing and prefer risky decisions. For example, Darke and Freedman (1997) found that individuals who held luck beliefs purchased more lottery tickets after experiencing a luck event, but individuals who did not hold luck beliefs purchased fewer lottery tickets after experiencing a luck event. Zhou et al. (2012) used a football lottery and found that luck had a positive effect on the frequency of lottery ticket purchases, with lottery players who believed in luck purchasing more tickets compared to those who believed in ability. Lim and Rogers (2017) also found that individuals who felt lucky purchased lottery tickets more frequently but also made more variety-seeking choices.

Consequently, consumers who feel lucky believe that they have a supportive power and can use this power to control the outcome of events as well as their personal fate. Thus, they hold a positive mindset regarding various life events and become more accepting of unfamiliar products (Menon & Kahn, 1995; Fan & Jiang, 2017). Therefore, consumers who feel lucky tend to exhibit more variety-seeking.

**Hypothesis 1**: Luck perception positively influences consumer variety-seeking.

Research on luck suggests that consumers who feel lucky become more energetic and possess more preferences for unfamiliar or novel choices. For example, consumers who feel lucky are more likely to use online banking, rather than a physical bank, to deposit money (Jiang et al., 2009). In financial markets, consumers who feel lucky have a higher interest in sudden and unfamiliar market information; they are more willing to pay for random shock signals in the lottery market (Gao et al., 2021). It is evident that luck motivates consumers and develops their interest in and desire for novel and unknown things. Novelty seeking is the innate tendency of consumers to show pleasure or excitement for novel stimuli (Thomas-Walters, 2021).

Based on the above analysis, we argue that consumers who feel lucky possess a strong motivation to pursue novelty.

The motivation for novelty seeking is one of the important factors influencing individual behavior. It represents consumers' innate tendency to exhibit pleasure or excitement towards novel stimuli, and serves as one of the driving forces for consumers to actively pursue new experiences (Nguyen et al., 2020). This is manifested in two aspects: intrinsic novelty seeking is the individual's desire for novel and exciting stimuli; extrinsic novelty seeking is manifested in the individual's actual behavior to obtain new stimuli. Therefore, we believe that the mechanism of luck’s influence on variety-seeking emerges from consumer novelty-seeking motivation. This is because variety-seeking is an expression of consumers’ search for novelty and pursuit of maximizing psychological utility, which can satisfy consumers’ novelty needs (Cao & Wang 2018). If consumers repeatedly choose a product, their stimulation for the product becomes weaker and fails to satisfy their novelty needs. on the other hand, found that when consumers have strong novelty-seeking motivation, they will change their routine purchasing behaviors, such as choosing new and unfamiliar products or brands. Meanwhile, when their novelty-seeking motivation is weaker, they will continue to maintain routine buying behavior (Sahni & Gupta, 2019).

In sum, luck perception can give consumers a power resource that enhances their novelty-seeking motivation, and variety-seeking can satisfy their novelty-seeking needs. Therefore, luck affects consumer variety-seeking, and novelty-seeking motivation is the mediating mechanism.

**Hypothesis 2**: Novelty-seeking motivation mediates the influence of luck perception on consumer variety-seeking.

4. Methodology
Comment: The explanation of structural equation modeling (SEM) is dense and may be difficult for readers unfamiliar with advanced statistical techniques. While the fit indices are reported, there is little explanation of what these indices mean or why they indicate an acceptable model fit. Additionally, the assumptions underlying SEM (such as measurement invariance and suppression effects) are mentioned but not clearly explained.
Recommendation: The methodology is detailed but dense, making it challenging for readers to follow.
**Revision note**: This paper adopts experimental methods, utilizing relatively simple variance analysis and regression analysis techniques. Structural equation modeling is not suitable for this paper, and therefore, there is no need to introduce structural equation modeling

5. Results
Comment: The results are statistically robust but lack sufficient interpretation.
Recommendation: Highlight the practical significance of findings, such as how marketers can leverage these insights to design interventions that enhance variety-seeking.
We have enriched the practical value of the paper

“Luck is increasingly being used by businesses as an actionable marketing element to influence consumer behavior. These firms want to influence consumers’ consumption decisions by improving their luck perception. Therefore, This paper’s findings have managerial implications for business managers or marketers.

Firstly, the findings have implications for firms expanding their product lines and launching new brands. When enterprises launch new products or brands, enterprise managers or marketers can consider altering consumers' luck perception to influence their consumption decisions. For instance, they can incorporate lucky numbers or symbols on product packaging or during advertising campaigns. When promoting products or brands offline, they can utilize lucky projection techniques or lucky draws to stimulate consumers' luck perception, fostering a greater variety of demands and enhancing their preference for new products or brands, thereby achieving market promotion for new products and brands.

Secondly, the research conclusions assist enterprises in implementing precise marketing. Enterprise managers or marketers can utilize big data technology, such as by simplifying the cognitive closure needs scale, to simply inquire about consumers' cognitive closure needs and identify their personality traits, thereby tracking and identifying consumers with high cognitive closure needs. When facing these consumers, enterprise managers or marketers can push luck-related information to them while they browse products, stimulating their sense of luck, increasing their variety-seeking behavior, and encouraging them to purchase more different products, thereby increasing the sales volume of the enterprise.”
6. Discussion
Comment: The discussion effectively summarizes the results but does not fully explore their theoretical and practical implications.
Recommendation: Provide a deeper analysis of how these findings contribute to understanding consumer psychology. Discuss potential applications in marketing and product design.
**Revision note**: We further explored the theoretical and practical implications of the research conclusions.

“Thirdly, this paper provides an in-depth analysis of self-concept change, which is the underlying mechanism influencing the impact of luck on consumer behavior. According to the positive self-concept explanation, luck perception may affect active self-perception, and this transient change in self-concept is the reason luck has an impact on individual behavior. For example, luck can influence individual behavior through changes in self-efficacy, sense of control, etc. This paper discovered that luck perception affects novelty-seeking motivation, changes in this novelty-seeking motivation affect consumer variety-seeking, and novelty-seeking motivation is the mechanism by which the sense of luck perception affects variety-seeking. Additionally, this paper found that luck positively affects consumers’ emotions, but these emotional changes are not the reason luck affects variety-seeking. This does not mean that positive emotions do not work, but the influence of emotions is more prevalent. This also validates Darke and Freedman’s (1997) hypothesis that luck causes individual to become more optimistic and does not affect their uncertainty behaviors but makes them rely more on familiar behaviors. Another possible explanation is that there may be an inverted U-shaped relationship between the effects of positive emotions on variety-seeking (Menon & Kahn, 1995). When positive emotions are strong, individuals’ variety-seeking decreases, leading to a non-significant effect of positive emotions on variety-seeking. By excluding the effect mechanism of emotion and confirming the effect mechanism of novelty-seeking motivation, we have provided an in-depth theoretical and empirical exploration of how the luck perception affects consumer variety-seeking and enriched the research on the mechanism of luck’s influence on consumer behavior.”
7. Conclusion and Future Directions
Comment: The conclusion is concise but could offer more specific suggestions for future research.
Recommendation: Suggest investigating cultural differences in luck perception or exploring its impact on other consumer behaviors. Discuss how longitudinal studies could extend these findings.

**Revision note**: We have improved this part of the content.

“The findings of this paper are limited to the conclusions drawn from the experiments we conducted, and there are some limitations that provide many opportunities for future research.

This paper only tests the explanatory mechanism of novelty-seeking motivation in the effect of luck perception on variety-seeking. Other explanatory mechanisms could be considered in the future. In risky decision making, it has been demonstrated that control illusions and facilitation orientation can explain the role of luck in influencing risky behavior (Day & Maltby, 2003), but all these studies have validated luck’s influence from a cognitive perspective, whereas emotions have not been explored in depth, except the finding that emotions such as gladness and optimism do not explain the role of luck’s influence on some risky behaviors. This is only in the domain we considered, where the influence of luck-induced positive emotions on behavior was not evident. However, emotions may have significant effects on other behavioral domains. For example, positive affect increases individual’s social and benevolent behavior (Hamerman & Morewedge, 2015). Therefore, future research could verify whether luck perception and positive emotions have an impact on consumers’ helping behaviors.

Moreover, luck is a cultural phenomenon. In terms of luck priming effects, participants who chose a strong culture of luck had more pronounced effects than those who chose a less strong culture of luck (Jiang et al., 2009; Chen et al., 2020; Tao et al., 2023). Therefore, in addition to the influence of high need for cognitive closure traits on the luck priming effect, cultural differences–– luck priming’s suitability to consumers with Eastern cultural backgrounds or consumers in Western countries––is an equally important determinant of proneness to luck. Future research could examine how cultural differences influence luck and luck’s effects. and examine whether luck has an impact on cultural consumption.”

8. Final Comments (Minor Revision)
The manuscript addresses an important topic in consumer psychology and marketing. However, to enhance its clarity and impact, revisions are needed to elaborate on methodological details, deepen theoretical discussions, and explore practical implications. With these improvements, the study has the potential to make a significant contribution to the field.

**Revision note**: We elaborate on the research methodology throughout the entire paper, deepen theoretical discussions, and explore practical implications
